# ﻿*Neottiamaolanensis*, a replacement name for *Neottiabifida* M.N.Wang (Orchidaceae)

**DOI:** 10.3897/phytokeys.235.113651

**Published:** 2023-11-14

**Authors:** Mei-Na Wang, Xin-Yi Wu, Cheng-Jiang Tan, Ping Yu, Wen-Hui Rao, Jie-Shan Chen, Jian Li, Jian-Bing Chen

**Affiliations:** 1 The Orchid Conservation & Research Center of Shenzhen and the National Orchid Conservation Center of China, Shenzhen Key Laboratory for Orchid Conservation and Utilization, Key Laboratory of National Forestry and Grassland Administration for Orchid Conservation and Utilization, Shenzhen, 518114, Guangdong, China The Orchid Conservation & Research Center of Shenzhen and the National Orchid Conservation Center of China Shenzhen China; 2 Management Department of Maolan National Nature Reserve, Libo 558400, Guizhou, China Management Department of Maolan National Nature Reserve Libo China

**Keywords:** homonym, illegitimate, *Neottiamaolanensis*, Orchidaceae, replacement name

## Abstract

According to Articles 53.1 of the International Code of Nomenclature for Algae, Fungi, and Plants (Shenzhen Code), *Neottiabifida* M.N.Wang (as 'bifidus'; PhytoKeys 229: 222, 2023) is an illegitimate name, and hence a new name *Neottiamaolanensis* M. N. Wang is proposed here.

A new mycoheterotrophic orchid species was named as *Neottiabifidus* M. N. Wang in our recent work (Wang et al. 2023). However, it was overlooked that the specific epithet spelled as “bifidus” is incorrect. The correct one should be “*bifida*”. Unfortunately, we found “*Neottiabifida*” is a later homonym (Blume 1825). According to Articles 6.11, 53.1 of the International Code of Nomenclature for Algae, Fungi, and Plants (Turland et al. 2018) that is currently in force, a new name is proposed here.

## ﻿ Neottia maolanensis

Taxon classificationPlantaeAsparagalesOrchidaceae

M.N. Wang nom. nov.

89FFC695-876F-5E8E-BB1E-C47295E814D4

urn:lsid:ipni.org:names:77330789-1

### Replaced name.

*Neottiabifida* M.N.Wang (as ‘*bifidus*’; in PhytoKeys 229: 222, 2023), *nom. illeg*., non. *Neottiabifida* Blume (in Bijdr. Fl. Ned. Ind. 8: 408, 1825).

### Type.

China. Guizhou Province, Qiannan Buyi and Miao Autonomous Prefecture, Libo County, the Maolan National Nature Reserve, 825 m elev., 23 Apr 2021, J.B.Chen 00599 (holotype: NOCC!).

### Chinese name.

茂兰鸟巢兰.

### Etymology.

The species epithet refers to the type locality of the new species.

## Supplementary Material

XML Treatment for Neottia maolanensis

## ﻿Additional information

### Author ORCIDs

Mei-Na Wang https://orcid.org/0000-0001-5998-6028

Xin-Yi Wu https://orcid.org/0000-0002-8623-1867

Cheng-Jiang Tan https://orcid.org/0009-0008-4558-4907

Ping Yu https://orcid.org/0009-0003-3319-7145

Wen-Hui Rao https://orcid.org/0000-0002-2177-6700

Jie-Shan Chen https://orcid.org/0009-0000-8859-6053

Jian Li https://orcid.org/0000-0002-0096-6257

Jian-Bing Chen https://orcid.org/0000-0001-9085-0663

### Data availability

All of the data that support the findings of this study are available in the main text.
